# The Latest “Cure” for Consumption

**Published:** 1892-03

**Authors:** 


### EDITORIAL DEPARTMENT.

F. E. DANIEL, M. D., EDITOR AND PUBLISHER.

THIS JOURNAL, by unanimous vote of the members, was made the Official Organ of the Austin District Medical Society.

COLLABORATORS:

*Dr. R. M. Swearingen, Austin.*
*Prof. B. E. Hadra, M. D., Galveston.*
*Prof. Geo. Cupples, M. D., San Antonio.*
*Dr. T. C. Osborn, Cleburne, Texas.*
*Dr E. J. Doering, Chicago.*
*Dr. E. J. Beall, Fort Worth.*
*Dr Odo Betz, Germany.*
*Prof. W. B. Rogers, M. D., Memphis.*
*Dr. J. W. McLaughlin, Austin.*
*Dr. T. J. Tyner, Austin.*
*Prof. J. F. Y. Paine, M. D., Galveston.*
*Dr. R. H. L. Bibb, Mexico.*
*Dr. T. J. Bennett. Austin.*
*Dr. Bat Smith, Wharton.*
*Dr. E. Meierhof, New York.*
*L. H. Luce, M. D., West Tisbury, Mass.*

### THE LATEST "CURE" FOR CONSUMPTION.

And now comes Prof. W. R. Amick, of Cincinnati, "Professor of Ophthalmology and Otology," etc., etc., who, following in the footsteps of the illustrious Koch, announces that *he* has discovered a sure cure for consumption (*and* asthma) and who, also like Koch,—"ain't agoin' " to tell you what it is. He saw with what avidity the profession took Koch's announcement *on faith*, —and contrary to every principle of medical ethics used the "lymph," without having the faintest conception of what it was (yet knowing it was deadly),—and now makes bold to announce *his* cure. "My chemical treatment" he calls it, without deigning to say what it is, further than it "consists of three parts; medicine put up in the form of tablets; second, medicine in a fluid form; and third, an inhaler and medicine for the inhaler." He explains, "the tablets are for supplying or increasing the elements that are wanting" [?]—"the drops are to allay the irritative and inflammatory tendency [?] and build up or develop the resisting force of the constitution, [?] the inhaler is for the purpose of allaying the inflammation and irritation in the lungs, and relieve the cough."—(*Lancet Clinic.*) [Here we remark, parenthetically, if the "inhaler" is for that purpose, it is a mechanical and not a "chemical" cure.]

This is all very fine in theory, and as complicated as it is fine. It must indeed be a wonderful "medicine put up in tablets" or "medicine in a fluid form" that will do all this; and it would be interesting to know what it consists of. But that Prof. Amick doesn't propose to tell us; that's his secret; that's where the money comes in, and that's where the gullibility comes in, and yet Prof. Amick is a college professor. Have the ethics of medicine gone to the bow-wows?

With such illustrious examples before him as Koch and Keely [?] it is not to be wondered at that Prof. Amick keeps secret his alleged discovery. But it is most surprising that the staunch and ethical *Lancet Clinic* should have been taken in by such clap-trap, and should lend its pages—*twenty-two columns*—to advertising Amick and his *nostrum*. Even should it have been paid for, it is a matter of great surprise to those who have watched the *Lancet Clinic* under the ethical and dignified management of Culberson to see such stuff paraded in its front pages.

And, it seems, from Amick's publication (March, ibid) of letters from doctors, and professors at that, that already the gullible profession have begun to fall in with his scheme, and to use his "tablets and drops" (without an idea of what they contain). Some of the "testimonials" there published are of the stereotyped newspaper kind, and only lack the addition of the words, "$1.00 a bottle, for sale by all druggists," to make them complete; for instance, here are a few testimonials given in full:

*Case II.*—Wm. Kemery. Previous duration of disease, three years. Symptoms: cough, moist rales, with free expectoration, loss of flesh, strength and appetite, night-sweats, hectic fever, pinched features, pain in the lungs, and dullness in upper portioon of both lungs. Began treatment October 22, 1891. Duration of treatment, two months. Result: complete recovery.

*Case XIV.*—Mrs. P—, aged 25, married. In December, 1890, and January, 1891, I attended her for a severe attack of pneumonia with a slow but fair recovery. In December, 1891, she caught a severe cold, and her lungs became rapidly involved. There was hoarseness, cough, night-sweats, loss of flesh and appetite, and great loss of strength. I immediately placed her upon the chemical treatment, and at the present writing, February 17, 1892, her recovery has been all that I could ask.

*Case XIX.*—James P., aged 38, married, bricklayer. Is subject to frequent colds, and has had catarrh, bronchial irritation with a chronic cough for months. He was placed upon the chemical treatment and he appeared infatuated with it, as he said "it reached the seat of the disease." Result, recovery.

ASTHMA.

*Case XXIX.*—Mrs. M., aged 59. Asthma and bronchial cough. She was placed on the chemical treatment for asthma and completely relieved in one week. No return of any asthmatic symptoms since.

*Case XXX.*—Mrs. Z., aged 43. Asthmatic history. Has had asthma for three years. She was placed upon the chemical treatment December 16, 1891. The husband reported that she did not suffer any more with asthma after the first night.

*Case XXXI.*—Fred. G., aged 36. On December 16, 1891, he had a severe attack of bronchial asthma. He was placed upon the chemical treatment at once with almost instant relief, and has had no more attacks.

*Case XXXII.*—Mr. W. had a severe attack of bronchial asthma. He is of a consumptive habit. He was placed upon the chemical treatment for asthma with the very best of results.

And so on, *ad nauseam*. *Forty* of these *things* are paraded in the *Lancet Clinic* to boost up Dr. Amick's "chemical treatment" —a *nostrum* in the fullest sense. The JOURNAL challenges anyone to produce a viler piece of quackery and effrontery. How like the stereotyped form in all newspapers,—"I was one mass of sores. Doctors could do me no good; was given out by the ablest professors. I heard of your wonderful Golden Medical Royal Radical Compound and bought a bottle. I am now sound and well. 50c. and $1 a bottle. Sold by all druggists."

Can such things be? We certainly are very nearly ruined by cheeky charlatans *in* the profession.

Prof. Amick should get out an *Almanac:* that is the place for such testimonials; and we suggest, he could then put pictures in, of "before taking and after taking." Shame!

